# Changes in health expenditures in China in 2000s: has the health system reform improved affordability

**DOI:** 10.1186/1475-9276-12-40

**Published:** 2013-06-13

**Authors:** Qian Long, Ling Xu, Henk Bekedam, Shenglan Tang

**Affiliations:** 1 School of Public Health and Management, Chongqing Medical University, Chongqing, P.R. China; 2 Center for Health Statistics and Information, Ministry of Health, Beijing, P.R. China; 3 World Health Organization, Western Pacific Region Office, Manila, Philippines; 4 Duke Global Health Institute, Duke University, Durham, NC, USA

**Keywords:** Health expenditures, Financial burden, Rural population, China

## Abstract

**Background:**

China's health system reform launched in early 2000s has achieved better coverage of health insurance and significantly increased the use of healthcare for vast majority of Chinese population. This study was to examine changes in the structure of total health expenditures in China in 2000–2011, and to investigate the financial burden of healthcare placed on its population, particularly between urban and rural areas and across different socio-economic development regions.

**Methods:**

Health expenditures data came from the China National Health Accounts study in 1990–2011, and other data used to calculate the financial burden of healthcare were from China Statistical Yearbook and China Population Statistical Yearbook. Total health expenditures were divided into government and social expenditure, and out-of-pocket payment. The financial burden of healthcare was estimated as out-of-pocket payment per capita as a percentage of annual household living consumption expenditure per capita.

**Results:**

Between 2000 and 2011, total health expenditures in China increased from Chinese yuan 319 to 1888 (United States dollars 51 to 305), with average annual increase of 17.4%. Government and social health expenditure increased rapidly being 22.9% and 18.8% of average annual growth rate, respectively. The share of out-of-pocket payment in total health expenditure for the urban population declined from 53% in 2005 to 36% in 2011, but had only a slight decrease for the rural population from 53% to 50%. Out-of-pocket payment, as a percentage of annual household living consumption, has continued to rise, particularly in the rural population from the less developed region (6.1% in 2000 to 8.8% in 2011).

**Conclusions:**

The rapid increase of public funding to subsidize health insurance in China, as part of the reform strategy, did not mitigate the out-of-pocket payment for healthcare over the past decade. Financial burden of healthcare on the rural population increased. Affordability among the rural households with sick members, particularly in the less developed region, is getting worse. It needs effective measures on cost control including healthcare provider payment reform and well developed health insurance schemes to offer better financial protection for the vulnerable Chinese seeking essential healthcare.

## Introduction

China has been facing substantial challenges to tackle increased inequality in health improvement, and access to healthcare between urban and rural areas and across different socio-economic development regions since its transformation from the planned economy to the market-oriented one [1]. The policy of cost recovery via revenues generated from service charges, which has been implemented in the public health sector of China particularly since early 1980s, has resulted in the poor efficiency of the Chinese health system in providing its population with equitable and affordable healthcare [2]. Since then, the central government had shrunk its investment in health. Health facilities including public hospitals have gained increasing financial autonomy to generate revenues and manage surpluses, combined with the introduction of bonus systems for the staff [3]. Meanwhile, the privatization of many state-owned enterprises and the disappearance of collective communes in the rural areas led to a dramatic decrease in health insurance coverage, leaving over half of the urban population and 90% of the rural population uninsured in 1990s. User fees for services and medicines became the major sources of financing for healthcare [4]. Changes in health financing introduced in the 1980s have led to rapid cost escalation of healthcare in China. As a consequence, the number of patients, mostly in rural areas and low income quartile, either did not seek care at all, or had informal care, or delayed and discontinued seeking care due to difficulty in affording healthcare [5-7]. High out-of-pocket payment for healthcare also placed heavy financial burden on many households, resulting in short- or long-term negative social and economic consequences, and even constituting the trap of poverty [7,8].

In response to increased concerns of affordability for healthcare, the Chinese government re-established rural health insurance scheme (called new rural cooperative medical scheme – NCMS) in 2003, and developed the urban resident basic medical insurance (URBMI) in 2007, with substantial financial subsidies from central and local governments and modest premium contributions from individuals, while strengthening urban employee basic medical insurance (UEBMI), a social health insurance scheme jointly contributed by employees and employers in urban China [9]. In 2009, the central government of China further announced a comprehensive reform of the health system, focusing on expanding health insurance coverage, establishing an essential medicine program, financing of public health interventions and primary healthcare, and piloting public hospital reform. The government has committed to spending additional Chinese yuan (CN¥) 850 billion (about United States dollars, US$ 125 billion) in 2009–2012 with an ultimate goal of achieving universal coverage of basic healthcare [10].

The new round of health system reform in China has been evolving rapidly. By the end of 2011, there have been several achievements emanating from the health system reform. Among them are over 90% of the Chinese population was covered by three above-mentioned health insurance schemes and significant increases in the use of healthcare in general and the reduction of inequality in access to healthcare between the poorest and wealthiest economic groups and across regions [11]. However, studies on financial protections of healthcare by these health insurance schemes reported mixed results [10-12]. It has not been clear that the new health system reform with almost universal coverage achieved in China has improved financial affordability of the Chinese people in seeking healthcare. Against this background, this study was to examine changes in the structure of total health expenditures in China in 2000–2011, and to investigate the financial burden of healthcare placed on its population, particularly between urban and rural areas and across different socio-economic development regions. The paper tries to answer if financial affordability for healthcare in China has been improved via the rapid population coverage of government supported health insurance schemes developed in the recent decade.

### Data and methods

Data on health expenditures in China in 2000–2011 was obtained from the China National Health Accounts Report 2012 published recently. In the report, data on health expenditure disaggregated by province were available only for 13 out of 31 provinces. Due to lack of disaggregated data on health expenditures or/and population by province and geographic location (e.g. urban vs. rural) in selected years, we can only manage to undertake appropriate analysis of data of the 13 provinces in the period from 2005 to 2011. Those were therefore included in this study to compare the structure of health expenditure and financial burdens across different socio-economic development areas of China over time. Data for the report was derived from the China national health accounts study between 1990 and 2011 conducted by the China National Health Development Research Centre (former China Health Economics Institute). The study included information on the structure of total health expenditures.

The indicators used in this study were total health expenditure per capita, disaggregated into government health expenditure, social health expenditure and out-of-pocket payment. In the report, government health expenditure refers to expenditures incurred by the central and local government authorities, including spending on healthcare and population and family planning, subsidy to health insurance, and health administration costs; social health expenditure refers to expenditures incurred by social funds, including spending on social health insurance, private health insurance, and social-medical assistance and donation, and administration cost. Out-of-pocket payment per capita was calculated as the sum of total expenditure of the urban population and total expenditure of the rural population divided by the number of population. The expenditure of the urban population included expenses on healthcare and health related products purchased. For the rural population, it was only healthcare expenses. In addition, out-of-pocket payment per capita as a percentage of average annual household living consumption expenditure per capita which defines as total consumption expenditure for daily life including food, clothing, housing, education, healthcare, transportation etc., was used as a proxy indicator to estimate the financial burden of healthcare placed on population.

The number of the population, stratified for the urban and rural population, and average annual household living consumption expenditure per capita in 2000–2011 were obtained from the China statistical yearbook and China population statistical yearbook. Gross Domestic Products (GDP) per capita in 2011 was calculated and used to classify the 13 study provinces´ level of development as developed (GDP per capita > CN¥50,000 including Beijing, Shanghai, Tianjing, Zhejiang and Guangdong), less developed (GDP per capita CN¥30,000-50,000 including Shandong, Fujian, Jilin and Heilongjiang), and least developed (GDP per capita < CN¥30,000 including Xinjiang, Jiangxi, Yunnan and Gansu) (CN¥ 1 = US$ 0.15). Over the period from 2000 to 2011, the ranks of study provinces´ development level were not changed much.

Changes in total health expenditures per capita, including government health expenditure, social health expenditure and out-of-pocket payment, were described over time and compared between urban and rural areas, and across different socio-economic development regions. Out-of-pocket payment as a percentage of average annual household living consumption expenditure of the urban and rural population nationwide and across different socio-economic development region in 2000–2011, was calculated respectively.

## Results

### Health expenditures

**Figure 1:**
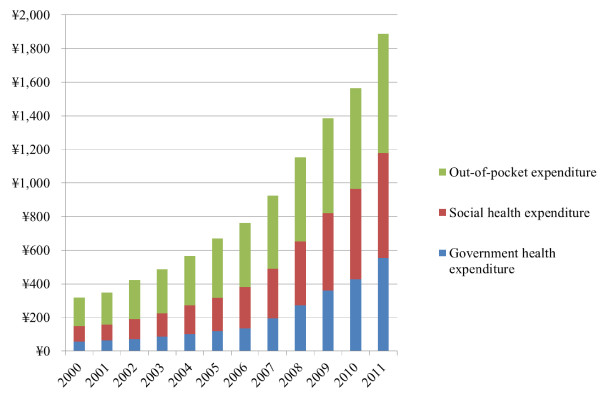
Total health expenditure per capita (Chinese yuan) in China in 2000-2011.

Total health expenditure per capita in China rose from CN¥319 in 2000 to CN¥1888 in 2011, with average annual increase of 17.4% (Figure 1). Between 2000 and 2011, government and social health expenditure grew rapidly, which on average increased 22.9% annually from CN¥56 to CN¥554, and 18.8% from CN¥92 to CN¥625, respectively. Out-of-pocket payment increased less substantial annually, from CN¥171 in 2000 to CN¥710 in 2011. The share of out-of-pocket payment in total health expenditure decreased over time, from 53% in 2005 to 38% of total health expenditure in 2011.

**Figure 2:**
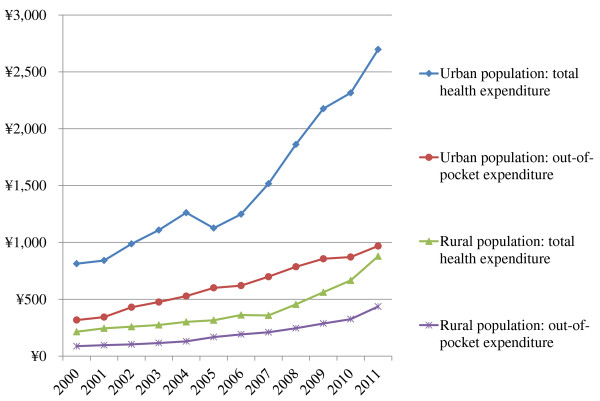
Total and out-of-pocket payment per capita (Chinese yuan) by the urban and rural population in 2000-2011.

Generally speaking, the total health expenditure of the urban population was much higher than that of the rural population in each year (Figure 2). For both the urban and rural population, the greatest increase in total health expenditure was seen from 2005 onwards. In 2011, the amount for the urban population (CN¥2698) was 3 times higher than that of the rural population (CN¥879). Between 2000 and 2011, the out-of-pocket payment increased 10.5% annually among the urban population and 15.6% among the rural population, respectively. The out-of-pocket payment as percentage of total health expenditure was 50% for the rural population in 2011, compared to 36% for the urban population.

**Figure 3:**
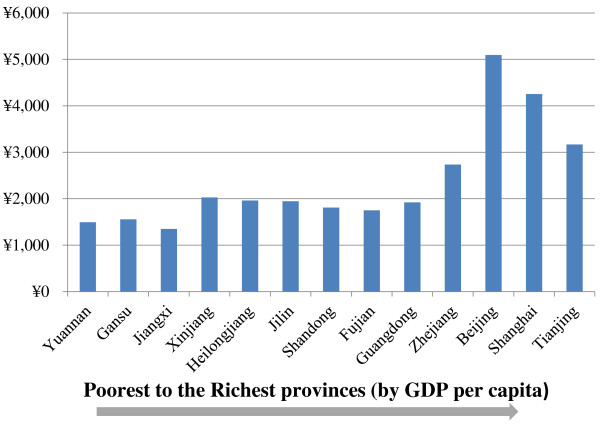
Total health expenditure (Chinese yuan) by 13 Chinese provinces ranked by GDP per capita in 2011.

Among the 13 study provinces ranked by GDP per capita, the total health expenditure increased with economic prosperity (Figure 3). In 2011, the total health expenditure was 1.8 times difference between the developed and least developed regions, and was about 3 times difference between the two richest municipalities (Beijing and Shanghai) and the two poorest provinces (Yunnan and Gansu).

Between 2005 and 2011, the government and social health expenditure per capita grew faster in the least and less developed regions than in the developed region (Table 1). In 2011, the government health expenditure in the least developed region was higher than in the less developed region, and the difference of this expenditure between the least developed and developed region was smaller. The difference in social health expenditure across regions remained high in 2011, however. There was 2.5 times difference between the least developed and developed region, and 1.8 times difference between the less developed and developed region.

**Table 1:** Total health expenditure (Chinese yuan) per capita by socio-economic development regions between 2005 and 2011

	**2005 (CN¥)**			**2011 (CN¥)**			**Average annual increase (%), 2005-2011**		
	**Least**	**Less**	**Developed**	**Least**	**Less**	**Developed**	**Least**	**Less**	**Developed**
Total health expenditure	484	669	1319	1544	1845	2751	+21.8	+18.8	+13.3
a)Government health expenditure	116	104	209	577	481	657	+31.4	+29.7	+21.5
(a)% of total health expenditure)	(23.9)	(15.5)	(15.8)	(37.4)	(26.1)	(23.9)			
b)Social health expenditure	109	174	466	449	614	1106	+27.2	+23.9	+15.8
(b)% of total health expenditure)	(22.5)	(26.0)	(35.3)	(29.1)	(33.2)	(40.2)			
c)Out-of-pocket payment	259	390	645	518	751	988	+12.5	+11.8	+7.5
(c)% of total health expenditure)	(53.5)	(58.3)	(48.9)	(33.5)	(40.7)	(35.9)			

Over the study period, the out-of-pocket payment increased greater in the least and less developed regions than in the developed region (Table 1). In 2011, the share of out-of-pocket payment in the total health expenditure was 41% in the less developed region, which was higher than in the least and developed region.

### Financial burden

Between 2000 and 2011, the out-of-pocket payment per capita as a percentage of average annual household living consumption expenditure per capita increased too (Table 2). For the urban population, this proportion increased from 6.4% in 2000 to 7.6% in 2005, and then gradually decreased to 6.4% in 2011. For the rural population, the out-of-pocket payment as a share of average annual household living consumption expenditure continued to increase from 5.2% in 2000 to 8.4% in 2011.

**Table 2:** Out-of-pocket payment per capita as a percentage of average annual household living consumption expenditure per capita (%) for the urban and rural population and across socio-economic development regions in 2000-2011

	**2000**	**2001**	**2002**	**2003**	**2004**	**2005**	**2006**	**2007**	**2008**	**2009**	**2010**	**2011**
Urban												
Nationwide	6.4	6.5	7.1	7.3	7.4	7.6	7.1	7.0	7.0	7.0	6.5	6.4
Least developed	5.7	-	-	-	-	7.5	7.0	6.7	6.6	6.7	6.1	6.6
Less developed	6.8	-	-	-	-	8.0	7.5	7.5	7.4	7.6	6.9	6.7
Developed	5.6	-	-	-	-	6.8	6.4	6.0	6.0	5.9	5.4	5.4
Rural												
Nationwide	5.2	5.6	5.7	6.0	6.0	6.6	6.8	6.5	6.7	7.2	7.4	8.4
Least developed	5.0	-	-	-	-	6.8	6.7	6.6	6.7	7.2	7.1	8.0
Less developed	6.1	-	-	-	-	7.1	7.2	6.7	7.2	7.6	8.1	8.8
Developed	5.2	-	-	-	-	6.7	6.6	6.0	6.5	6.5	6.8	7.7

Note: Data for the years of 2001–2004 were not available in these study provinces.

Similar trends for both the urban and rural population were seen in all three different socio-economic development regions (Table 2). In 2011, in the less developed region the out-of-pocket payment accounted for higher percentage of annual household living consumption expenditure of both the urban and rural population than those in the least developed and developed regions. Among two of the four provinces grouped into the less developed region, the out-of-pocket payment was over 10% of annual household consumption expenditure of its population, reaching a threshold of catastrophic expenditure [13-15].

## Discussion

Since early 2000s the Government of China has been tackling the issue of inequity in access to, and financing of, healthcare. As a result, there has been a great increase in the total health expenditure with larger shares of government and social health expenditure. There have been many positive developments toward universal health coverage in China: rapid expansion of health insurance coverage; increased access to, and use of, healthcare; the introduction of essential medicine programme etc. [16]. Nevertheless, we found that the out-of-pocket payment for healthcare remained high. Financial burden of healthcare placed on individual households continued to increase, especially in rural areas and the less developed region.

This study is based on the national health accounts data to describe a trend in public and private health spending in general and across different socio-economic development regions. While there are several limitations related to an estimation of financial burden of healthcare. The national health accounts are aggregate data of general population. It is not able to address demographic distribution and whether people seeking healthcare or not. Thus out-of-pocket payment per capita as a share of average annual household living consumption expenditure per capita can not accurately reflect affordability of healthcare. It was used as a proxy indicator in this study and was found increasing over time. This may suggest an increased financial risk, particularly for households with sick members, and have a set of research and policy implications.

Over the past decade, the rapid increase in healthcare costs may be well associated with rising quantity of service uses, particularly inpatient services. According to the Chinese health statistical digest [17], the numbers of outpatient visits and inpatient admissions in China had increased from 2.12 billion to 3.45 billion, and from 52.97 million to 132.56 million, respectively, over the period from 2000–2009. The increase of public spending in healthcare was expected to encourage the provision of responsive and quality healthcare, and reduce inefficiencies of service delivery. Around half of the government health investment is used to subsidize the health insurance schemes, and the rest is used for the construction of health facilities, the hiring of qualified staffing, and the compensation of preventive institutions [10]. Coinciding with the development of health insurance and improvement of availability of healthcare, the utilization of healthcare has greatly increased in 2003–2011 [11]. The increase in hospital admission in rural areas was particularly striking, as the benefit packages of the NCMS in most places of China primarily cover inpatient services. This is not a surprising finding, as many international studies reported the positive relationship between health insurance coverage and use of health services [18-20].

Over-provision of healthcare, owing to either perverse financial incentive to health service providers or/and increased demand for high quality care, also fuels the growth of healthcare costs as well as negatively impacts on efficiency of healthcare delivery associated with potential adverse health outcomes. Fee-for-service payment has been embedded in the Chinese health system since the health sector reform launched in 1980s. The income of service providers is tied up the revenue generated for facilities through a bonus system [3]. As a result, service providers become less conscious of cost, and opted for more and more expensive procedures. In addition, almost a universal coverage of health insurance may encourage service users to take unnecessary diagnostic tests and treatments, a moral hazard occurred when people are covered by health insurance. In rural China, the total expenditure of facility-based baby delivery between 2002 and 2007 doubled, accompanied with a notable increase of caesarean section rate from 13% to 26% [12]. The study in five rural counties in central and western provinces reported that three fourth of caesarean section was performed as non-emergency caesarean section. The NCMS was associated with having a caesarean section, particularly non-emergency caesarean section due to both doctors´ recommendation and women's request by themselves [21,22]. In the meantime, a lack of effective referral system implemented in the Chinese health systems has also resulted in a fact that an increasing number of service users had bypassed primary healthcare facilities to seek healthcare at higher level hospitals. The health statistics show that the proportion of outpatient visits and inpatient admissions to township health centers reduced from 39.5% to 26.6%, and from 32.3% to 29.2%, respectively, in 2000–2009 [17].

The health system reform unveiled in 2009 has introduced a few strategies to control healthcare costs, as the cost escalation of healthcare is often a great challenge in financing of affordable healthcare in almost all the countries. One of the strategies is the implementation of an essential medicine list. A number of provincial/municipal health authorities in China have developed a policy that does not allow their service providers to put any mark-up rate on essential medicines (namely zero mark-up) when they sell medicines to service users [23]. Such a policy intended for cost control of healthcare, but is not welcomed by many service providers and the pharmaceutical industry. In 2011, the Ministry of Health reported that there were half public health facilities not adopting the essential medicine list [16]. It may suggest a continued profit-driven drug prescription under the ‘zero-mark-up’ drug policy. One recent study that compared the behaviour of prescription between the rich and poor cities found that in the rich cities, health facilities prescribed fewer but more expensive drugs, while, in the poor cities, they prescribed more drugs, even if per prescription was less expensive [24]. The cost of healthcare is affected by the price of services/goods and the volume of services/goods consumed. China has been focusing more on the issue of price, such as price of medicines, than the volume of services provided, when tackling the rapid rise of healthcare costs. At the provincial level of China, there is a department of pricing that has an authority to approve the pricing of many services and goods including health services and pharmaceutical products. Controlling the prices of medicines has not been approved as a successful means for healthcare cost containment in China, as the service providers often increased the quantity of service provision, while the Chinese pharmaceutical manufactures stopped the production of medicines whose production profits have once reduced significantly. Therefore, it would have to be imperative for the Chinese government to remove the perverse incentives currently given health service providers through a number of effective measures, such as developing alternative provider payment methods. That will have to be a key to controlling of healthcare cost and the improvement of quality care in order to ensure the success of the reform in a long term.

Uncontrolled growth of cost and limited benefits of the insurance schemes has resulted in high out-of-pocket payment on the patients, particularly from the rural areas of China. As mentioned previously, NCMS’s service benefit package often, focuses on paying for inpatient care. Those suffering from non-communicable chronic diseases (NCDs), such as hypertension and diabetes, and requiring outpatient services usually paid fully or in a large proportion, for outpatient service expenses out of pocket [25]. In 2010, many counties start including outpatient care, such as major NCDs into the NCMS benefit package [10]. However, a previous study in Shandong and Ningxia found a very low reimbursement level for outpatient care ranging from less than 1% to 13% [26], mainly depending on capacity of financial mobilization for the health insurance schemes. Even for inpatient services, patients with the NCMS coverage have to pay over half of medical costs [10,16]. In our study period in 2000–2011, the financial burden of healthcare on general rural population has continued to increase, accounting for 8.4% of average annual household living consumption expenditure in 2011. Affordability of healthcare for the households with patients, such as NCD patients would have to be worse. For the urban population, the out-of-pocket payment as a percentage of average annual household consumption expenditure decreased slightly in the study period. This was due largely to relatively better service benefit packages and financial protection offered by UEBMI and also probably URBMI [27]. In addition, the rapid increase of household consumption among the urban population has also been witnessed as the increase in average income per capita was faster among the urban population than among the rural population over the period of 2000–2011 (12.0% verse 10.7%), based on China statistical yearbook .

Across different socio-economic development regions, a heavier financial burden of healthcare placed on the population was seen in the less developed region, not in the least developed region. In China, the fiscal status of local governments in the western region of China was not good enough to allocate sufficient financial resources to support social services [28]. Therefore, the central government has allocated increasingly the earmarked fund for health investment, such as financial subsidies for the NCMS and support to the constructions and/or upgrading of primary healthcare facilities, to the poor western region through fiscal transfer policy [10]. This has significantly contributed to a fast increase in the government health expenditure of the least developed region. The less developed region has not received significant financial supports from the central government, while its fiscal status has often not been well able to provide adequate funding to support social developments including healthcare. In several relative poor provinces in the less developed region ranked by GDP per capita, the average out-of-pocket payment for healthcare was more than 10% of average annual household living consumption expenditure, which may indicate heavier burden on households with sick members, even constitute a great risk of medical impoverishment. Similar results have been reported in the paper by Meng and his colleagues, indicating that the rural households and households in the central region experienced catastrophic health expense more often than the urban households and households in the eastern region; while regional difference in having catastrophic health expense was smaller than rural–urban difference [11].

## Conclusions

China has increased substantially the government investment in health, particularly for the poor region, and subsidized health insurance schemes to achieve universal coverage, resulting substantial increases of utilization of health services for both inpatient and outpatient. The increase in public funding to subsidize health insurance, however, did not mitigate the out-of-pocket payment for healthcare and improve efficiency of service delivery. The financial burden of healthcare on the rural population, particularly from the less developed region, increased year by year in 2000s, showing a poorer affordability of healthcare.

Effective measures on cost control and better policies of health insurance schemes in China badly need to be developed in the near future to offer better financial protection for the vulnerable Chinese seeking essential healthcare. We recognize that the Government of China has already piloted a number of alternative provider payment methods to tackle the rapid rise of healthcare cost. It is important to scale up these effective measures aimed for cost control. New injection of public funding into healthcare in China should ensure the reduction of financial burden of healthcare on the population, improving the affordability of quality care, and achieving better health outcomes. That is what the new health system reform in China is aimed for.

## Abbreviations

GDP: Gross domestic products; NCDs: Non-communicable chronic diseases; NCMS: New (rural) cooperative medical scheme; URBMI: Urban resident basic medical insurance; UEBMI: Urban employee basic medical insurance.

## Competing interests

The authors declare that they have no competing interests.

## Authors’ contributions

ST contributed to the overall conceptualization, study design, interpretation of findings and the article writing. QL participated in the study design, conducted the analysis and drafted the paper. LX and HB participated in the interpretation of findings and commented on the article. All authors read and approved the final manuscript.
